# Clinical features and independent predictors of postoperative refractory trauma to anal fistula combined with T2DM: A propensity score-matched analysis-retrospective cohort study

**DOI:** 10.3389/fsurg.2023.1119113

**Published:** 2023-02-24

**Authors:** Xiao Tang, Taohong He, Xinyi Li, Ya Liu, Yuqi Wu, Gehang You, Jie Li, Yu Yun, Lei Wu, Li Li, Jian Kang

**Affiliations:** ^1^ Department of Proctology, Hospital of Chengdu University of Traditional Chinese Medicine, Chengdu, China; ^2^ School of Clinical Medicine, Chengdu University of Traditional Chinese Medicine, Chengdu, China; ^3^ Department of Endocrinology, Hospital of Chengdu University of Traditional Chinese Medicine, Chengdu, China

**Keywords:** anal fistula, type 2 diabetes mellitus, open trauma, wound healing, independent predictors, clinical features

## Abstract

**Background:**

Refractory wound is a common postoperative complication in anal fistula surgery, when combined with type 2 diabetes mellitus (T2DM) it presents a slower recovery time and more complex wound physiology. The study aims to investigate factors associated with wound healing in patients with T2DM.

**Materials and methods:**

365 T2DM patients who underwent anal fistula surgery at our institution were recruited from June 2017 to May 2022. Through propensity score-matched (PSM) analysis, multivariate logistic regression analysis was applied to determine independent risk factors affecting wound healing.

**Results:**

122 pairs of patients with no significant differences were successfully established in matched variables. Multivariate logistic regression analysis revealed that uric acid (OR: 1.008, 95% CI: 1.002–1.015, *p *= 0.012), maximal fasting blood glucose (FBG) (OR: 1.489, 95% CI: 1.028–2.157, *p *= 0.035) and random intravenous blood glucose (OR: 1.130, 95% CI: 1.008–1.267, *p *= 0.037) elevation and the incision at 5 o’clock under the lithotomy position (OR: 3.510, 95% CI: 1.214–10.146, *p *= 0.020) were independent risk factors for impeding wound healing. However, neutrophil percentage fluctuating within the normal range can be considered as an independent protective factor (OR: 0.906, 95% CI: 0.856–0.958, *p *= 0.001). After executing the receiver operating characteristic (ROC) curve analysis, it was found that the maximum FBG expressed the largest under curve area (AUC), glycosylated hemoglobin (HbA1c) showed the strongest sensitivity at the critical value and maximum postprandial blood glucose (PBG) had the highest specificity at the critical value. To promote high-quality healing of anal wounds in diabetic patients, clinicians should not only pay attention to surgical procedures but also take above-mentioned indicators into consideration.

## 1. Introduction

Anal fistula, mostly formed by inflammatory cells, collagen and epithelial tissue is a pathological channel between the anal or rectum and the skin (1). Its incidence fluctuates between 10.4/100,000 to 23.2/100,000 (2, 3), however, due to the privacy of the lesion site and the low consultation rate, the true incidence might be higher. The causes of anal fistulas are complex, but most of them are formed after the rupture of the perianal abscess which is caused by anal gland infection (4). Sometimes they are considered to be different stages of the same disease. The abundance of perianal connective tissue and tissue spaces can contribute to complex and variable anal fistula alignment. About 59.0%–71.0% of them are low anal fistulas, and 62.3%–67.0% of them are intersphincteric fistulas (5, 6). The nature of the anal fistula and the high cure rate have dictated that anal fistulectomy and cutting seton surgery are still the most commonly performed clinical procedures (7–9). However, after these procedures, surgeons must face the cruel fact that these wounds rely on a large amount of granulation tissue to fill the defects. Previous studies have found even in procedures with smaller trauma areas, wound recovery time remained long (10–12). It is crucial to reduce the effect of confounding factors by adjusting the baseline data.

When combined with other underlying diseases, the trauma-healing process is even lumpier. Patients with T2DM in developing countries, represented by China and India, are expected to increase by 69% between 2010 and 2030, reaching a staggering 693 million by 2045 (13–16). Diabetic patients are often associated with slow wound healing, and when it comes to wounds occurring in the dense anal nerve, it greatly increases patients’ pain and decreases their quality of life. As a complex recovery process, the circulatory metabolic state of the body could continuously influence wound healing by reshaping the internal environment. Elevated blood glucose affects wound healing process by upgrading inflammation levels, dysfunction oxidative stress, and slowing down angiogenesis (17–20). Previous reports of other surgeries reveal that abnormal blood glucose metabolism increases the risk of infection and impedes wound healing after other procedures (21, 22). However, there is a lack of studies on postoperative wound recovery in anal fistula patients with T2DM. This study included statistics of laboratory tests, surgical modalities, and postoperative treatment. The information gap was filled on anal fistula wound healing in T2DM patients. As a continuous and changing process, wound recovery is more complex and variable in diabetic patients. PSM is necessary to exclude some unpredictable confounding factors and logistic regression could be applied to target risk factors for wound healing. Meanwhile, the effect of blood glucose has been elaborated by using the receiver operating characteristic (ROC) curve. The study provides a therapeutic direction to promote rapid healing after anal fistula surgery.

## 2. Method

### 2.1. Diseases definition and data collection

The diagnosis of anal fistula is based on the German S3 guidelines: anal abscess and fistula (23). All patients were diagnosed with anal fistula by anal finger examination, anoscope examination, radiographic examination (including rectal endoluminal ultrasound, pelvic CT, or MRI), or intraoperative probe/methylene blue staining, and the number of internal orifices was counted by these techniques. The diagnostic criteria for T2DM were based on the latest Chinese guidelines for the prevention and treatment of T2DM set by the Chinese Diabetes Society (24, 25). And the diagnosis was assigned by an endocrinologist. Relevant data were collected on the cases, including demographic characteristics, clinical features, laboratory and ancillary tests at admission, anal fistula-related information (e.g., previous surgical history, anal fistula types, number of internal orifices, etc.), pre- and post-surgical treatments, and surgical modalities. Non-healing (refractory) group refers to trauma that cannot be repaired in time with conventional therapy or wounds that can not achieve functional recovery and anatomical integrity (26). The last routine dressing change time in the outpatient clinic was collected as the outcome indicator. Judged by the specialist anorectologist and the definition of the relevant literature, patients were divided into the non-healing (refractory) group or healing group according to whether its recovery period is longer than 35 days (27–29).

Among the underlying diseases, hypertensive disease and non-alcoholic fatty liver diseases are listed independently. Chronic cardiovascular diseases included coronary atherosclerotic heart disease and lacunar cerebral infarction. Chronic lung diseases included tuberculosis, chronic obstructive pulmonary disease, and chronic pulmonary heart disease. Chronic liver diseases included chronic viral hepatitis B, cirrhosis of the liver, hepatic hemangioma, etc.

### 2.2. Inclusion exclusion criteria and follow up

**Figure 1:**
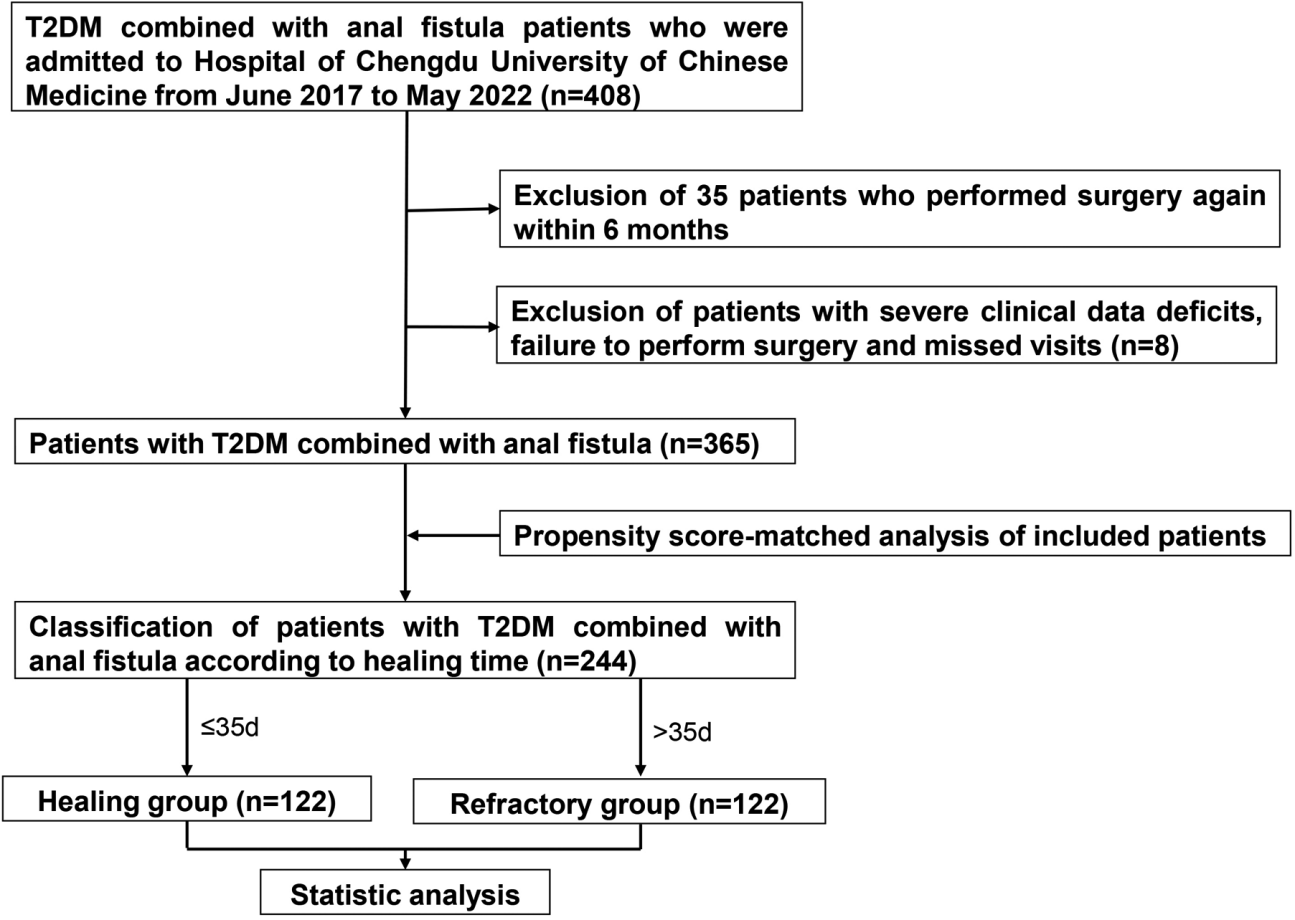
Research flow chart.

This clinical retrospective study collected 408 cases of anal fistula combined with T2DM attending the Hospital of Chengdu University of Chinese Medicine from June 2017 to May 2022. The study excluded anal fistula caused by Crohn's disease or exotic injuries (from indigestible diets or external devices). Extremely complicated fistulas that were not suitable for fistulotomy, cutting seton surgery or a combination procedure were screened out. And fistulas in the active phase of anal fistula were excluded. Research recruited 408 patients, but 43 patients were eliminated because of recurrence (within 6 months) (*n* = 35), severe clinical data deficits, failure to perform surgery, or missed visits. In the end, a total of 365 patients were included. Up on wound recovery time, patients were divided into the non-healing (refractory) group (*n* = 170) and the healing group (*n* = 195), respectively. After matching, there were 122 patients in each group. The detailed operation procedure was shown in Figure 1. This study was approved by the ethics committee of the Hospital of Chengdu University of Traditional Chinese Medicine (ethics number: 2022KL-018). The research was a retrospective clinical study and only the patients’ previous treatment data were extracted through the medical system. Therefore, no informed consent was required from the patients.

### 2.3. Statistical analysis

All statistical analysis were performed with SPSS version 22.0 (IBM, Armonk, NY, USA). Continuous variables were expressed as mean ± standard (SD) deviation or M (QL, QU). Before conducting propensity score, the student's *t*-test and Mann–Whitney *U* test were applied for continuous variables, whereas the *χ*^2^ test was used for dichotomous variables. To eliminate relevant confounders and increase comparability between different group, PSM analysis was performed using nearest-neighbor matching (1:1) adjusted for baseline data including: gender, age, body mass index (BMI), history of anal fistula surgery, the number of internal orifices (≥2 or not) and the number of wounds (≥3 or not), with the caliper value set at 0.02. After PSM, variables were tested by paired *t*-test and Wilcoxon rank sum test for continuous variables. McNemar test and Fisher's exact test for categorical variables. Risk factors for non-healing wound were identified by univariate and multivariate logistic regression models, and the degree of association was shown by calculating odds ratio (OR). Variance inflation factor (VIF) and tolerance were applied to determine the covariance between internal orifice and incision, as well as multicollinearity among variables before multivariate regression. To prevent overfitting, multiple variables with statistically different (*p* < 0.05) were selected for multivariate logistic regression analysis to identify independent predictors of healing trauma. Also, we used ROC curve to assess the sensitivity and specificity of glucose indicators in wound healing. All statistical tests were two-sided, and *p* < 0.05 was considered statistically significant. The experimental procedure of the article was carried out with reference to the relevant authoritative literature (27).

## 3. Results

### 3.1. Description of baseline data

The majority patients were male, reaching astonishing 347 cases (95.07%). The healing group had more female patients than the refractory group. However, there was no discernible difference between each other (*p* = 0.248). BMI (26.83 ± 0.33 vs. 26.97 ± 0.28 Kg/m^2^) and previous surgical history (19 vs. 21) in the healing group were slightly higher than those in the non-healing group. According to more than two types of ancillary findings, the number of patients with multiple internal orifices was larger in the healing group (*n* = 173, 88.72%), while cases with ≥3 wounds were more in the refractory group (42 vs. 89, *p* = 0.003). No significant difference was discovered between two groups in terms of underlying diseases (*p* > 0.05).

122 pairs of patients were successfully matched (Table 1) with no significant difference between gender (*p *= 1.000), age (*p *= 0.923), BMI (*p *= 0.547), previous anal fistula surgery history (*p *= 1.000), number of internal orifices (*p *= 1.000), and number of wounds (*p *= 1.000).

**Table 1:** The clinical characteristics of patients with anal fistula combined with T2DM.

Variables	Before PSM				After PSM^a^			
	Total (*n* = 365)	Refractory group (*n* = 175)	Healing group (*n* = 190)	*p* value^b^	Total (*n* = 244)	Refractory group (*n* = 122)	Healing group (*n* = 122)	*p* value^c^
**Gender, *n* (%)**				0.248				1.000
Male	347 (95.07%)	164 (96.47%)	183 (93.85%)		240 (98.26%)	120 (98.36%)	120 (98.36%)	
Female	18 (4.93%)	6 (3.53%)	12 (6.15%)		4 (1.64%)	2 (1.67%)	2 (1.67%)	
**Age (year)**	50 (40, 57)	50 (41, 59)	50 (44, 55)	0.779	48.54 ± 0.79	48.62 ± 1.17	48.45 ± 1.07	0.923
**BMI (Kg/m^2^)**	26.91 ± 0.21	26.83 ± 0.33	26.97 ± 0.28	0.744	27.05 ± 0.25	27.11 ± 0.41	26.98 ± 0.33	0.547
**Previous anal fistula surgery, *n* (%)**				0.901				1.000
Yes	40 (10.96%)	19 (11.18%)	21 (10.77%)		26 (10.66%)	14 (11.48%)	12 (9.84%)	
No	325 (89.04%)	151 (88.82%)	174 (89.23%)		218 (89.34%)	108 (88.52%)	110 (90.16%)	
**Number of internal orifices, *n* (%)**				0.003^*^				1.000
=1	304 (83.29%)	131 (77.06%)	173 (88.72%)		212 (86.89%)	106 (86.89%)	106 (86.89%)	
≥2	61 (16.71%)	39 (22.94%)	22 (11.28%)		32 (13.11%)	16 (13.11%)	16 (13.11%)	
**Number of wounds, *n* (%)**				<0.001^*^				1.000
<3	234 (64.11%)	81 (47.64%)	153 (78.76%)		160 (65.57%)	80 (65.57%)	80 (65.57%)	
≥3	131 (35.89%)	89 (52.35%)	42 (21.54%)		84 (34.43%)	42 (34.43%)	42 (34.43%)	
**Other comorbidities, *n* (%)**
Hypertensive disease	105 (28.77%)	54 (31.76%)	51 (26.15%)	0.238	76 (34.84%)	42 (34.43%)	34 (27.87%)	0.788
Non-alcoholic fatty liver diseases	235 (64.38%)	114 (67.06%)	121 (62.05%)	0.319	161 (65.98%)	79 (64.75%)	82 (67.21%)	0.807
Prostatic hyperplasia	61 (16.71%)	26 (15.29%)	35 (17.95%)	0.498	43 (17.62%)	19 (15.57%)	24 (19.67%)	0.188
Chronic pulmonary disease	10 (2.74%)	6 (3.53%)	4 (2.05%)	0.574	7 (2.87%)	4 (3.28%)	3 (2.46%)	0.727
Chronic liver disease	16 (4.38%)	6 (3.53%)	10 (5.13%)	0.610	10 (4.10%)	5 (4.10%)	5 (4.10%)	0.727
Cardiovascular diseases	15 (4.11%)	10 (5.88%)	5 (2.56%)	0.111	10 (4.10%)	5 (4.10%)	5 (4.10%)	1.000

^a^ Matching cohort information (gender, age, sex, BMI, history of anal fistula surgery, the number of internal orifices and the number of wounds) included in PSM.

^b^ Calculated using the *χ*^2^ test, student's *t*-test, or Mann–Whitney *U* test.

^c^ Calculated using paired *t*-tests, Wilcoxon rank sum test, or the McNemar test.

* Significant at *p* < 0.05.

### 3.2. Laboratory and ancillary tests

The results of the detailed laboratory and ancillary tests are presented in Table 2. The concentration of plasma sodium (137.45 mmol/L [135.78, 140.10] vs. 139.45 mmol/L [137.58, 140.83], *p* < 0.001) and plasma chloride (102.94 ± 0.34 vs. 103.94 ± 0.29 mmol/L, *p* = 0.039) showed a higher level in non-healing group. No significant abnormalities or intergroup differences were discovered in the rest of electrolytes such as potassium, calcium, magnesium and phosphorus in plasma. Uric acid, one of the key metabolic byproducts in the body, demonstrated an aberrant range in the non-healing group, where the level reached 359.50 (292.50, 433.50) mmol/L, compared to the healing group's 314.00 mmol/L (255.50.372.00). In terms of lipid metabolism, both groups displayed abnormal ranges in cholesterol (5.46 mmol/L [4.43, 6.25] vs. 5.16 mmol/L [4.37, 6.20]) and triglyceride (1.99 mmol/L [1.34, 2.78] vs. 2.30 mmol/L [1.67, 3.41]). However, no statistical significance (*p* > 0.05) was detected in cholesterol, triglyceride, high-density lipoprotein (LDL), low-density lipoprotein (HDL). Meanwhile, except for the A/G ratio (1.60 [1.40, 1.80] vs. 1.70 [1.50, 1.95], *p* = 0.041), the remaining indicators of albumin, globulin, alanine transaminase (ALT) and aspartate transaminase (AST) were not found to be different from the normal range or demonstrate differences between groups.

**Table 2:** Laboratory and ancillary tests on admission day.

Variables	Normal range	Total (*n* = 244)	Refractory group (*n* = 122)	Healing group (*n* = 122)	*p* value
Axillary temperature (°C)	36.0–37.1	36.4 (36.2, 36.7)	36.5 (36.2, 36.7)	36.4 (36.2, 36.6)	0.113
K (mmol/L)	3.5–5.3	4.11 (3.80, 4.45)	4.21 (3.83, 4.51)	4.02 (3.79, 4.36)	0.064
Na (mmol/L)	137–147	138.60 (136.90, 140.48)	137.45 (135.78, 14 0.10)	139.45 (137.58, 140.83)	<0.001^*^
Cl (mmol/L)	99–110	103.44 ± 0.23	102.94 ± 0.34	103.94 ± 0.29	0.039^*^
Ca (mmol/L)	2.11–2.52	2.32 (2.22, 2.40)	2.30 (2.21, 2.40)	2.33 (2.23, 2.42)	0.064
Mg (mmol/L)	0.75–1.02	0.82 ± 0.01	0.81 ± 0.01	0.82 ± 0.01	0.422
P (mmol/L)	0.85–1.51	1.10 ± 0.01	1.12 ± 0.02	1.09 ± 0.02	0.353
Blood urea nitrogen (mmol/L)	2.6–7.5	5.90 (4.66, 7.00)	5.87 (4.13, 6.91)	5.97 (5.00, 7.09)	0.079
SCR (mmol/L)	41–73	68.8 (61.00, 81.38)	69.70 (60.60, 84.93)	67.90 (61.98, 78.33)	0.467
Uric acid (mmol/L)	155–357	330.00 (274.00, 399.00)	359.50 (292.50, 433.50)	314.00 (255.50.372.00)	<0.001^*^
Random intravenous blood glucose (mmol/L)	3.93–6.11	10.50 (7.45, 15.33)	12.39 (8.27, 16.64)	8.88 (6.61, 12.41)	<0.001^*^
Albumin (g/L)	40–55	46.10 (43.23, 49.18)	46.05 (43.28, 49.00)	46.15 (43.20, 49.23)	0.751
Globulin (g/L)	20–40	28.20 (24.53, 32.20)	28.75 (25.00, 33.10)	27.45 (24.38, 31.35)	0.150
A/G	1.2–2.4	1.61 (1.47, 1.90)	1.60 (1.40, 1.80)	1.70 (1.50, 1.95)	0.041^*^
ALT (U/L)	7–40	27.00 (17.00, 44.75)	26.00 (17.00, 45.00)	28.50 (18.00, 44.25)	0.335
AST (U/L)	13–35	23.50 (18.00, 34.00)	23.00 (17.00, 34.00)	24.00 (18.00, 34.00)	0.960
GGT (U/L)	35–100	37.50 (24.00, 59.00)	38.50 (25.75, 59.00)	37.00 (22.75, 60.50)	0.490
Total bile acid (μmol/L)	0–10	4.80 (2.63, 7.50)	4.90 (2.48, 7.80)	4.75 (2.85, 7.50)	0.536
Total bilirubin (μmol/L)	0–21	14.50 (10.33, 20.35)	14.90 (10.25, 20.50)	13.85 (10.48, 19.30)	0.510
Cholesterol (mmol/L)	<5.18	5.20 (4.39, 6.21)	5.46 (4.43, 6.25)	5.16 (4.37, 6.20)	0.839
TG (mmol/L)	<1.7	2.09 (1.53, 3.10)	1.99 (1.34, 2.78)	2.3 (1.67, 3.41)	0.091
HDL (mmol/L)	>1	1.10 (0.93, 1.41)	1.09 (0.91, 1.57)	1.10 (0.93, 1.29)	0.168
LDL (mmol/L)	<3.3	2.70 ± 0.06	2.59 ± 0.08	2.78 ± 0.10	0.093
White blood cell count (×10^9^/L)	3.5–9.5	7.28 (5.84, 9.07)	7.39 (5.88, 9.15)	7.04 (5.77, 8.78)	0.493
Neutrophil count (×10^9^/L)	1.8–6.3	4.90 (3.66, 6.28)	4.86 (3.70, 6.30)	4.93 (3.64, 6.27)	0.803
Lymphocyte count (×10^9^/L)	1.1–3.2	1.67 (1.29, 2.16)	1.66 (1.35, 2.23)	1.70 (1.24, 2.14)	0.501
Monocyte count (×10^9^/L)	0.1–0.6	0.42 (0.3, 0.56)	0.45 (0.32, 0.56)	0.40 (0.29, 0.56)	0.397
NEUT%	40–75	67.30 (58.00, 74.20)	66.15 (54.78, 74.13)	67.80 (61.00, 74.88)	0.014^*^
LY%	20–50	26.20 (18.15, 47.33)	28.25 (18.00, 53.23)	26.20 (18.15, 47.33)	0.039^*^
MO%	3–10	7.35 (5.30, 29.53)	8.75 (5.60, 34.50)	7.35 (5.30, 29.53)	0.008^*^
Red blood cell count (×10^9^/L)	3.8–5.1	5.06 ± 0.03	5.07 ± 0.04	5.05 ± 0.05	0.838
Hemoglobin (g/L)	115–160	152.24 ± 0.98	152.00 ± 1.33	152.48 ± 1.44	0.754
RDW-SD (fl)	36.4–46.3	41.30 (38.90, 43.38)	40.75 (37.88, 42.80)	42.15 (39.58, 43.75)	0.001^*^
PLT (×10^9^/L)	100–300	211.14 ± 3.78	215.22 ± 5.39	207.25 ± 5.33	0.322
C-reactive protein (mg/L)	0–5	11.66 (3.91, 24.03)	13.15 (4.89, 30.45)	9.86 (2.51, 21.63)	0.064
Glycosylated hemoglobin (%)	4.1–6.1	8.10 (6.93, 9.70)	8.80 (7.68, 10.03)	7.40 (6.50, 8.73)	0.001^*^
Maximum FBG (mmol/L)	3.9–6.0	8.00 (6.90, 9.80)	9.00 (7.50, 10.40)	7.35 (6.70, 8.25)	<0.001^*^
Maximum PBG (mmol/L)	7.8–11.1	14.50 (12.80, 16.60)	15.15 (13.20, 17.53)	14.05 (12.28, 15.53)	<0.001^*^
Urine glucose	Negative				0.203
Normal		60 (24.59%)	25 (20.49%)	35 (28.69%)	
Abnormal		184 (75.41%)	97 (79.51%)	87 (71.31%)	
Urine ketone	Negative				0.008^*^
Normal		190 (77.87%)	85 (69.68%)	105 (86.07%)	
Abnormal		54 (22.13%)	37 (30.33%)	17 (13.93%)	
Endoanal ultrasound, *n* (%)		143 (58.61%)	71 (58.20%)	72 (59.02%)	1.000
Detection of ultrasound	Positive	136 (55.74%)	68 (55.74%)	68 (55.74%)	1.000

* Significant at *p* < 0.05.

Anal fistula lesions are confined by collagen and epithelial tissue and the infection is mostly under control. The results showed that white blood cell count (WBC) and neutrophil percentage (NEUT%) fluctuated in the normal range in both groups, while C-reactive protein (CRP) was higher than normal range. The NEUT% in the healing group reached 67.80% (61.00, 74.88) was greater than in non-healing group [66.15% (54.78, 74.13), *p *= 0.014]. At the same time, lymphocyte percentage (28.25% [18.00, 52.23] vs. 26.20% [18.15, 47.33], *p *= 0.039) and monocyte percentage (8.75% [5.60, 34.50] vs. 7.35% [5.30, 29.53], *p *= 0.008) in refractory group also exhibited a superior level. 143 patients carried out anorectal endoscopic ultrasonography, and the positive detection of fistulas internal orifices in the implemented patients reached 94.44%.

**Figure 2:**
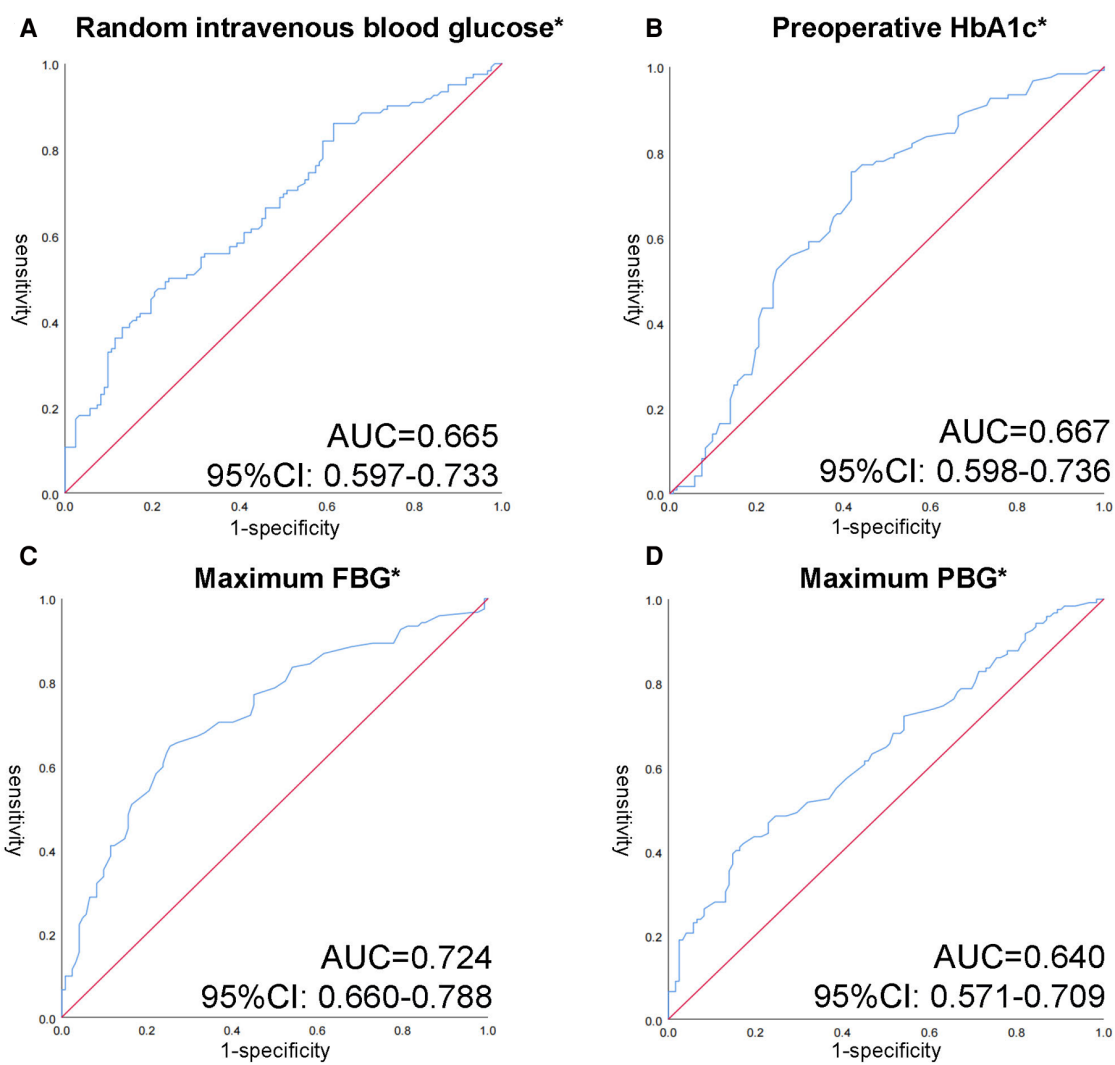
Receiver operating characteristic (ROC) curves of different glycemic indicators in patients with anal fistula and T2DM. (**A**) ROC curve of random intravenous blood glucose. (**B**) ROC curve of preoperative HbA1c. (**C**) ROC curve of maximum FBG. (**D**) ROC curve of maximum PBG. *p < 0.05 with Wilcoxon rank sum test.

It exhibits a clear distinction between glucose indicators. It is worth mentioning that random intravenous blood glucose (tested with other biochemical indicators) and HbA1c were extracted preoperatively. The finger-prick glucose test was used to monitor FBG and PBG. Patients in the healing group had lower random intravenous blood glucose (8.88 mmol/L [6.61–12.41]) compared to 12.39 mmol/L (8.27–16.64) in refractory group, with a *p* value less than 0.001. In response to medium and long-term blood glucose levels, HbA1c levels at admission and maximum PBG, and FBG levels throughout hospitalization has been collected. We noticed that HbA1c indicated a higher standard in non-healing group (8.80 mmol/L [7.68, 10.03] vs. 7.40 mmol/L [6.50, 8.73], *p *= 0.001). During the hospitalization, patients’ maximum FBG and the maximum PBG respectively reached 9.00 mmol/L (7.50, 10.40) and 15.15 mmol/L (13.20, 17.53) in the refractory group. Striking statistical differences between the two groups were shown (*p* < 0.001). Among the glucose related indicators, the area under the ROC curve of maximum FBG reached 0.724, followed by HbA1c 0.667, random blood glucose 0.665 and maximum PBG 0.640 (Figure 2). The critical values of HbA1c, random blood glucose, maximum FBG and maximum PBG were 7.69%, 12.50, 8.35 and 16.05 mmol/L, respectively. The sensitivity of each index at the critical values was 75.4%, 50.0%, 64.8%, and 41.8%, and the specificity was 58.2%, 76.2%, 74.6%, and 82.8%, respectively.

### 3.3. Surgery-related treatment results

Of these 244 patients, 40.98% (*n* = 100) were diagnosed with high anal fistula which patients in the refractory group was slightly higher than in the healing group (*n* = 56 vs. *n* = 44). Different surgical techniques were carried out by the surgeons depending on the type of fistula. 30 (12.30%) patients underwent cutting seton surgery, 144 (59.02%) patients performed anal fistulectomy and 70 (28.69%) patients underwent both procedure (hybrid surgery). Fisher's exact test did not reveal any statistical difference either overall or between the two different procedures (*p *> 0.05). In order to explore the effects of different incisions in wound healing, we summarized the characteristics of the distribution of wounds under lithotomy position. 6 o’clock incision was chosen most (*n* = 123, 50.41%), followed by the 3 o’clock incision (*n* = 64, 26.23%), 5 o’clock incision (*n* = 60, 24.59%), and 1 o’clock incision (*n* = 53, 21.27%). Both 1 o’clock and 5 o’clock wounds exhibited a higher incidence in the non-healing group and there were statistical differences in both groups (*p* = 0.005, *p* = 0.029). Milligan-Morgan surgery were conducted in 70 patients (28.69%) at the same time, while in the healing group this proportion only reached 25 cases (20.49%). The full surgery-related data are shown in Table 3.

**Table 3:** Surgery-related treatment results.

Variables	Total (*n* = 244)	Refractory group (*n* = 122)	Healing group (*n* = 122)	*p* value
**Hospital day (day)**	8 (7, 10)	8 (7, 10)	8 (7, 9)	0.104
**Incision before surgery, *n* (%)**				0.344
Yes	12 (4.92%)	4 (3.28%)	8 (6.56%)	
No	232 (95.08%)	118 (96.72%)	114 (93.44%)	
**Anal fistula type, *n* (%)**				0.104
High anal fistula	100 (40.98%)	56 (45.90%)	44 (36.07%)	
Low anal fistula	144 (59.02%)	66 (54.10%)	78 (63.93%)	
**Surgery method, *n* (%)** ^a^				0.416
Anal fistulectomy	144 (59.02%)	67 (54.92%)	77 (63.11%)	Anal fistulectomy vs. Cutting seton surgery: 0.324^a^
Cutting seton surgery	30 (12.30%)	13 (10.66%)	17 (13.93%)	Cutting seton surgery vs. Hybrid surgery: 0.501^a^
Hybrid surgery	70 (28.69%)	32 (26.23%)	38 (31.15%)	Anal fistulectomy vs. Hybrid surgery: 0.179^a^
**Position of incision, *n* (%)**				
1 o'clock incision	53 (21.72%)	36 (29.51%)	17 (13.93%)	0.005^*^
2 o'clock incision	21 (8.61%)	11 (9.02%)	10 (4.92%)	1.000
3 o'clock incision	64 (26.23%)	32 (26.23%)	32 (26.23%)	1.000
4 o'clock incision	16 (6.56%)	8 (6.56%)	8 (6.56%)	1.000
5 o'clock incision	60 (24.59%)	38 (31.15%)	22 (18.03%)	0.029^*^
6 o'clock incision	123 (50.41%)	60 (49.18%)	63 (51.64%)	0.791
7 o'clock incision	43 (17.62%)	19 (15.57%)	24 (19.67%)	0.500
8 o'clock incision	14 (5.74%)	5 (4.10%)	9 (7.38%)	0.424
9 o'clock incision	44 (18.03%)	25 (20.49%)	19 (15.57%)	0.430
10 o'clock incision	18 (7.38%)	7 (5.74%)	11 (9.02%)	0.424
11 o'clock incision	48 (19.67%)	28 (22.95%)	20 (16.39%)	0.268
12 o'clock incision	14 (5.74%)	7 (5.74%)	7 (5.74%)	1.000
**Milligan-Morgan surgery, *n* (%)**				0.006^*^
Yes	70 (28.69%)	45 (36.89%)	25 (20.49%)	
No	174 (71.31%)	77 (63.11%)	97 (79.51%)	

^a^ Calculated using Fisher's exact test.

* Significant at *p* < 0.05.

### 3.4. Pre- and post-operative treatment

Treatment for anal fistula and T2DM must be administrated simultaneously when they are both present. To highlight the relationship between various therapies and wound healing, we gathered several interventions and results presented in Table 4. Among these patients, 92 (37.70%) patients had their first T2DM diagnosis with no statistically significant difference (*p *= 0.665). Insulin pumps were used for glycemic control in 61 (25.00%) patients during hospitalization, including 41 (33.61%) in the refractory group and 20 (16.39%) in the healing group. Among the patients who used them, the duration did not show a difference (6.49 ± 0.39 d vs. 5.00 ± 0.64 d, *p *= 0.053). There was no significant difference between the two groups regarding the adoption of subcutaneous insulin, but it did show a higher utilization rate on oral hypoglycemics in the healing group (71 vs. 90, *p *= 0.015).

**Table 4:** The treatment of anal fistula combined with T2DM.

Variables	Total (*n* = 244)	Refractory group (*n* = 122)	Healing group (*n* = 122)	*p* value
**Previous diagnosis of T2DM, *n* (%)**				0.665
Yes	92 (37.70%)	57 (46.72%)	35 (28.69%)	
No	152 (62.30%)	65 (53.28%)	87 (71.31%)	
**Usage of the insulin pump, *n* (%)**				0.002^*^
Yes	61 (25.00%)	41 (33.61%)	20 (16.39%)	
No	183 (75.00%)	81 (66.39%)	102 (83.61%)	
**Duration of insulin pump use (day)**	6.02 ± 0.34	6.49 ± 0.39	5.00 ± 0.64	0.053
**Oral hypoglycemic, *n* (%)**				0.015
Yes	161 (65.98%)	71 (58.20%)	90 (73.77%)	
No	83 (34.02%)	51 (41.80%)	32 (26.23%)	
**Subcutaneous Insulin Injections, *n* (%)**				0.519
Yes	48 (19.67%)	26 (21.31%)	22 (18.03%)	
No	196 (80.33%)	96 (78.69%)	100 (81.97%)	
**Preoperative antibiotic therapy, *n* (%)**				<0.001^*^
Yes	159 (65.16%)	65 (53.28%)	94 (77.05%)	
No	85 (34.84%)	57 (46.72%)	28 (22.95%)	
**Amount of antibiotics, *n* (%)**				0.839
1	219 (89.75%)	109 (89.34%)	110 (90.16%)	
2	25 (10.25%)	13 (10.66%)	12 (9.84%)	
**Duration of antibiotic use after surgery (day)**	5 (5, 7)	5 (5, 7)	5 (5, 7)	0.140
**Usage of polymyxin B, *n* (%)**				0.134
Yes	100 (40.98%)	44 (36.07%)	56 (45.90%)	
No	144 (59.02%)	78 (63.93%)	66 (54.10%)	

* Significant at *p* < 0.05.

In 244 cases, the rate of preoperative antibiotics reached 65.16%, with a higher proportion in the refractory group. The duration of postoperative intravenous antibiotics usage (5d [5, 7] vs. 5d [5, 7], *p *= 0.14) did not demonstrate a significant difference. 89.75% (*n* = 219) of the patients used only one antibiotic and 10.25% (*n* = 25) used two or more antibiotics after operation, no significant difference was seen between the groups (*p *> 0.05).

### 3.5 Results of univariate and multivariate logistic regression analysis

After the univariate logistic regression analysis of statistically significant indicators, all outcomes revealed various degrees of associations with wound healing with the exception of LY% (Table 5). Among the laboratory indices, elevated plasma sodium, chloride, A/G ratio, and NEUT% showed varying degrees of protection. Random intravenous blood glucose (OR: 1.137, 95% CI: 1.072–1.206, *p *< 0.001), HbA1c (OR: 1.284, 95% CI: 1.104–1.494, *p *= 0.001), maximal FBG (OR: 1.581, 95% CI: 1.1.313–1.904, *p *< 0.001) and excessive levels of maximal PBG (OR:1.182, 95% CI: 1.079–1.296, *p *< 0.001) were shown to be impairing factors for wound healing. Intriguingly, the usage of insulin pump had an OR > 1 in the univariate logistic regression analysis, whereas oral hypoglycemic drugs unsurprisingly played a protective role (OR = 0.50). In surgical treatment, 1 and 5 o’clock incision and concurrent M-M surgery both served to retard wound healing.

**Table 5:** Univariate regression analysis for relevant variables.

Variables	Univariate OR	95% CI	*p* value
Na	0.824	0.734–0.913	<0.001^*^
Cl	0.931	0.869–0.998	0.043^*^
Uric acid	1.005	1.004–1.006	<0.001^*^
Random intravenous blood glucose	1.137	1.072–1.206	<0.001^*^
A/G	0.412	0.197–0.880	0.022^*^
NEUT%	0.974	0.952–0.998	0.030^*^
LY%	1.010	0.998–1.022	0.090
MO%	1.021	1.005–1.038	0.011^*^
RDW-SD	0.869	0.799–0.945	0.001^*^
Glycosylated hemoglobin	1.284	1.104–1.494	0.001^*^
Maximum FBG	1.581	1.313–1.904	<0.001^*^
Maximum PBG	1.182	1.079–1.296	<0.001^*^
Urine ketone	2.500	1.280–4.883	0.007^*^
Usage of insulin pump	3.000	1.467–6.137	0.003^*^
Oral hypoglycemic	0.500	0.288–0.867	0.014^*^
Preoperative antibiotic therapy	0.318	0.174–0.518	<0.001^*^
Placement of drainage tube	8.000	1.001–63.962	0.050^*^
1 o'clock incision	2.583	1.327–5.030	0.005^*^
5 o'clock incision	2.000	1.097–3.645	0.024^*^
Milligan-Morgan surgery	2.429	1.303–4.525	0.005^*^

* Significant at *p* < 0.05.

To prevent overfitting, the research included plasma sodium, chloride, uric acid, random intravenous blood glucose, HbA1c, maximum FBG, maximum PBG, insulin pump usage and oral hypoglycemic drug use, 1 and 5 o’clock incision into the multivariate analysis by referring relevant literature and clinical experience. The results of multivariate logistic regression analysis are presented in Table 6. It revealed that the decrease of NEUT% (OR: 0.906, 95% CI: 0.856–0.958, *p *= 0.001), elevation of uric acid (OR: 1.008, 95% CI: 1.002–1.015, *p *= 0.012), maximum FBG (OR:1.489, 95% CI:1.028–2.157, *p *= 0.035), random intravenous blood glucose (OR: 1.130, 95% CI: 1.008–1.267, *p *= 0.037) and incision at 5 o’clock (OR: 3.510, 95% CI: 1.214–10.146, *p *= 0.020) were independent risk factors for refractory wounds.

**Table 6:** Multivariate regression analysis for relevant variables.

Variables	Multivariate OR	95% CI	*p* value	
NEUT%	0.906	0.856–0.958	0.001^*^	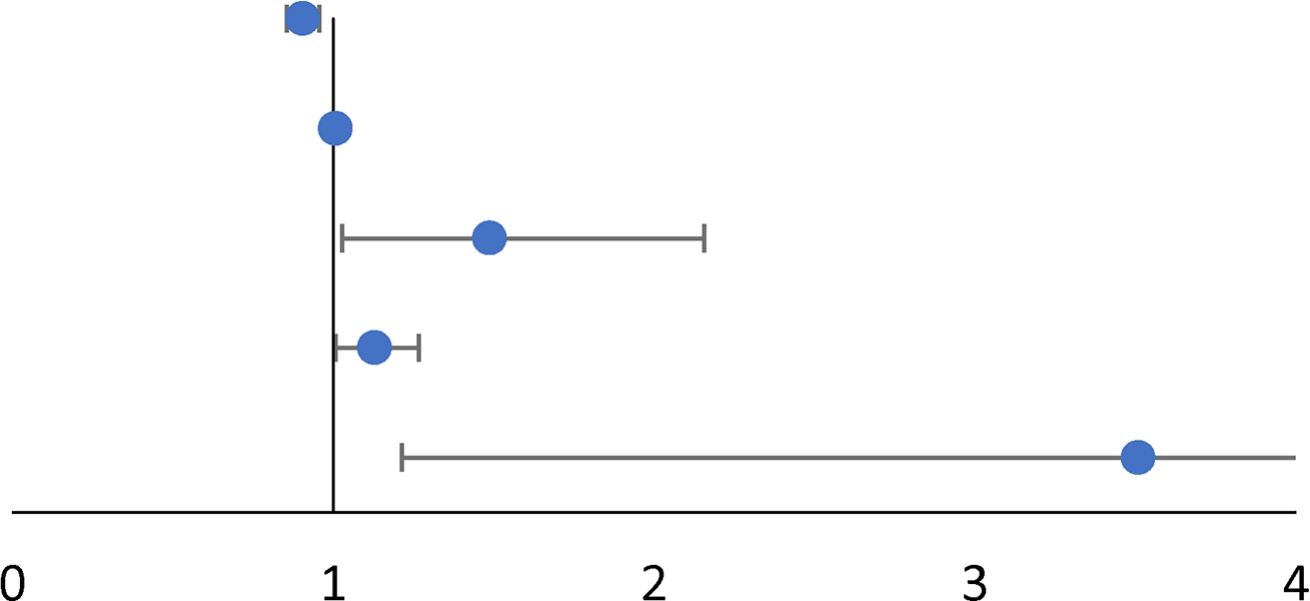
Uric acid	1.008	1.002–1.015	0.012^*^	
Random intravenous blood glucose	1.130	1.008–1.267	0.037^*^	
Maximum FBG	1.489	1.028–2.157	0.035^*^	
5 o'clock incision	3.510	1.214–10.146	0.020^*^	

* Significant at *p* < 0.05.

## 4. Discussion

It is widely accepted that surgical treatment is the only way to cure anal fistula. Surgical methods such as ligation of intersphincteric fistula tract (LIFT), video-assisted anal fistula treatment (VAAFT), mucosal advancement flap surgery, and biomaterial occlusion have developed rapidly (5, 6, 9). Fistulas fistulotomy and cutting seton surgery can fulfill general anal fistula treatment needs and are still the most commonly used procedure in clinical practice (7–9). However, due to the special physiological functions of the anus, the failure rate of anal fistula surgery reached 3.90%–31% (30, 31). Excessive healing time after anal fistula surgery is an important cause of surgical failure. It has been proved that the choice of surgical approach (32), increase of systemic inflammation (33) are risk factors for refractory wounds. In this research, the fistulas fistulotomy and cutting seton surgery do not show a difference in wound healing, this may indicate that infection played a more important role in wound healing than the trauma area, which fits with Ho KS's report (7). The key point of anal fistula surgery is the proper drainage of secretions. We found trauma at 5 o’clock is an independent risk factor for healing. Because the trauma located on the posterior side of the anal may constantly face large impact from defecation and leftover stool tended to penetrate from the 6 o'clock incision resulting in inefficient diversion effects.

Most purulent material has been drained by external orifices, infection has been limited in anal fistula patients (33). It is reasonable that WBC and NEUT% fluctuated within the normal range. Previous reports indicated that persistently elevated levels of inflammation at the trauma surface could delay trauma recovery, especially in diabetic patients (34). By multifactorial logistic regression, however, we found that acceptable evaluation of NEUT% favors wound healing. Unfortunately, this only represents the preoperative result, inflammation indicators after the surgery was unable to be collected, so the correlation between it and wound recovery can not be elucidated yet.

Successful surgery is the first step in securing wound healing, but the dynamics of the trauma environment will constantly affect wound healing. Mina Sarofim (30) reported that diabetes mellitus was an important risk factor for the recurrence of anal fistula. In this study, we found that elevated maximal FBG and RBG were independent risk factors for impeding wound healing. Escalated blood glucose delays wound healing in many ways. First and foremost, it increases the level of inflammation and prolongs the duration of infection. The persistent infiltration of inflammatory cells in the hyperglycemic state secretes large amounts of pro-inflammatory factors, aggravating the traumatic inflammation (35). Long glycolysis time increases neutrophil (36) and monocytes accumulation (37) and also slows down macrophage cell transformation, thus increasing secretion of cytotoxic substances. In addition, it's found that patients with refractory trauma exhibited higher levels of serum uric acid. Abnormalities in uric acid metabolism laterally contribute to the rise in inflammation. Previous studies have already suggested that inflammatory macrophage phenotype persistence in T2DM wounds leads to derangement catabolic process of uric acid, the retention of uric acid may lead to its crystallization in wounds, thus increasing the inflammation level (38, 39).

Secondly, chronic hyperglycemia status in T2DM impaired wound redox response, and excessive production of reactive oxygen (ROS) decreases the quality of wound healing. Toll-like receptor expression is upregulated, and hypoxia-inducible factors (HIFs) destabilization cause the imbalance of redox responses (24). In contrast, an oxygen-rich environment accelerates the survival and migration of keratin-forming cells and fibroblasts, thus promoting trabecular vascular growth (40). Third, the high glycemic state prevents the growth of blood vessels and the migration of newborn cells. Persistent hyperglycemia impaired epithelial and macrophage function reduced expression of growth factors and weakened pro-angiogenic signaling. The decline in growth factors directly impairs the proliferation, migration and differentiation processes of keratin-forming cells and fibroblasts, damaging body's ability to repair traumas (41). After ROC curve analysis, maximum FBG shows the largest AUC (0.724), which has the strongest predictive effect for postoperative wound healing. Although HbA1c has the strongest sensitivity at the critical value, it mainly reflects the blood glucose status before surgery. Patients intervened by professional endocrinologists after admission which made the blood glucose fluctuation within the hospital more in line with the long-term situation.

Hyperinflammatory, hypoxic, and disorders of angiogenesis formed an interactive network that feeds back to each other and restrains trauma healing. Well-managed blood sugar promotes high-quality wound healing. Patients with an insulin pump have an increased risk of non-healing trauma, which is not consistent with previously reported results (42). The main reason is that in clinical practice, insulin pumps were mostly used in patients with high blood glucose levels. From long-term perspective, these patients had worse glucose metabolism status. The critical solution is to optimize glucose metabolism to avoid delaying wound healing.

In general, wound healing is a multifactorial repair process. This study reports independent factors affecting wound healing in patients with anal fistula combined with T2DM. Most of the risk factors had a strong connection with inflammation and glycolysis. Uric acid, blood glucose and maximum fasting blood glucose elevation, and incision at 5 o’clock are independent risk factors for impeding wound healing. Elevated NEUT% within normal levels is a protective factor for trauma healing. We recommend that physicians actively monitor these indicators to ensure rapid wound healing. However, it has to be mentioned that there is still lack of RCT trials to support the certainty of its risk factors. Also, as a retrospective study, selection bias is inevitable, although we used PSM to minimize bias as much as possible.

## Data Availability

To protect patients' privacy, raw study data are available from the authors upon request.
